# Genetic dissection of QTLs for oil content in four maize DH populations

**DOI:** 10.3389/fpls.2023.1174985

**Published:** 2023-04-12

**Authors:** Xiaolei Zhang, Min Wang, Haitao Guan, Hongtao Wen, Changzheng Zhang, Changjun Dai, Jing Wang, Bo Pan, Jialei Li, Hui Liao

**Affiliations:** ^1^ Quality and Safety Institute of Agricultural Products, Heilongjiang Academy of Agricultural Sciences, Harbin, Heilongjiang, China; ^2^ National Maize Improvement Center of China, College of Agronomy and Biotechnology, China Agricultural University, Beijing, China; ^3^ Maize Yufeng Biotechnology LLC, Beijing, China; ^4^ Food Processing Institute, Heilongjiang Academy of Agricultural Sciences, Harbin, Heilongjiang, China

**Keywords:** Maize, DH, kernel, oil, QTL

## Abstract

Oil is one of the main components in maize kernels. Increasing the total oil content (TOC) is favorable to optimize feeding requirement by improving maize quality. To better understand the genetic basis of TOC, quantitative trait loci (QTL) in four double haploid (DH) populations were explored. TOC exhibited continuously and approximately normal distribution in the four populations. The moderate to high broad-sense heritability (67.00-86.60%) indicated that the majority of TOC variations are controlled by genetic factors. A total of 16 QTLs were identified across all chromosomes in a range of 3.49-30.84% in term of phenotypic variation explained. Among them, six QTLs were identified as the major QTLs that explained phenotypic variation larger than 10%. Especially, *qOC-1-3* and *qOC-2-3* on chromosome 9 were recognized as the largest effect QTLs with 30.84% and 21.74% of phenotypic variance, respectively. Seventeen well-known genes involved in fatty acid metabolic pathway located within QTL intervals. These QTLs will enhance our understanding of the genetic basis of TOC in maize and offer prospective routes to clone candidate genes regulating TOC for breeding program to cultivate maize varieties with the better grain quality.

## 1 Introduction

The modern maize (*Zea mays* L.) kernels are composed of approximately 72% starch, 10% protein, 4% oil, and 14% other constituents (Laurie et al., 2004; Ranum et al., 2014). Oil predominantly accumulates in the embryo and is stored in the form of triacylglycerols, which is composed of roughly 59% polyunsaturated, 24% monounsaturated and 13% saturated fatty acid (Dupont et al., 1990; Lambert, 2001). The proper ratio of unsaturated to saturated fatty acids in maize oil is considered as a character of high-quality oil for human health (Han et al., 1987; Benitez et al., 1999; Lambert et al., 2004). In addition, the high energy and proportion of polyunsaturated fatty acids is highly valued for animal feed, industrial applications and an alternative to fossil fuels (Hou et al., 2022). Thus, the ability to improve oil quantity and quality has been a key target for plant breeding and biotechnology-assisted improvement (Yang et al., 2012; Li et al., 2013).

High-oil maize hybrids (oil concentration > 6%) are considered as an important crop with valued nutrient (Wei et al., 2009). A series of genetic resources have been generated by long-term artificial selection of high-oil maize populations (Fang et al., 2021). The oil concentration of initial open-pollinated variety Illinois High Oil (IHO) reached about 20% after 100 generations of selection (Dudley and Lambert, 2004). A normal maize synthetic Zhongzong No. 2, which was synthesized with 12 inbred lines of Lancast heterotic group, was used to produce the Beijing High Oil (BHO) with oil concentration increased from 4.71 to 15.55% after 18 selection cycles (Song and Chen, 2004). The inbred line By804 was derived from the high-oil population ‘Beinongda’ and its oil concentration reached 11.22% (Zhang et al., 2008).

As the unique and precious resources, these high oil materials provide an opportunity to understand the genetic architecture of oil and fatty acid biosynthesis, which in turn increase the efficiency of selection to improve oil concentration and quality (Wassom et al., 2008a; Wassom et al., 2008b; Yang et al., 2010; Li et al., 2020). Combined with map-based cloning, QTL mapping is the most powerful and efficient strategy to identify the genomic region that controls complex quantitative traits in plants (Goldman et al., 1994; Lima et al., 2006; Messmer et al., 2009). The total oil content (TOC) is a quantitative trait, and many quantitative trait loci (QTL) have been demonstrated to control the seed oil accumulation in a randomly mated F_2:3_ population IHO × ILO (Alrefai et al., 1995; Berke and Rocheford, 1995; Laurie et al., 2004; Clark et al., 2006; Dudley, 2008). These studies revealed that TOC was controlled by numerous genes with individually small effects and mainly additive gene action (Yang et al., 2010). In addition, using a recombinant inbred line (RIL) population derived from B73 × By804, a relatively small number of QTL were detected and accounted for a large percentage of the total phenotypic variation (Song and Chen, 2004; Zhang et al., 2008; Yang et al., 2010; Pan et al., 2012; Yang et al., 2012). These studies also indicated that epistasis is a key factor affecting the genetic basis of oil content in maize kernel (Wassom et al., 2008b; Yang et al., 2010). Similar results were also obtained in two publicly available maize genetic resources, NAM (the nested association mapping population) and AMP508 (association mapping population) based on high-resolution and high power QTL analysis (Lambert et al., 2004; Cook et al., 2012). A high-oil QTL (*qHO6*) on chromosome 6 has been cloned and the candidate gene encodes an acyl-CoA:diacylglycerol acyltransferase (DGAT1-2), which catalyzes the final step of oil synthesis (Zheng et al., 2008). The major QTL QTL-*Pal9* explaining 42% of the phenotypic variation in palmitic acid content was identified on maize chromosome 9 in a bi-parental segregating population and the candidate gene *Zmfatb* encodes acyl-ACP thioesterase (Li et al., 2011).

Distinct mapping populations were featured with advantages and limitations, which results in significant impacts on QTL outputs (Odell et al., 2022). DH segregating populations have been commonly used in QTL analysis for several specific advantages (Chaikam et al., 2019). Complete homozygosity of DH lines allows accurate phenotyping over multiple locations and years compared to families in early selfing generations (Foiada et al., 2015; Yan et al., 2017). In this study, we utilized four DH populations derived from the practical breeding program to further dissect the genetic basis and QTLs controlling the phenotypic variation of TOC in maize kernels. Our intention was to describe the genetic architecture of oil variation in extensive scale and provide the prospective targets to identify candidate genes for increasing oil concentration in commercial maize germplasms.

## 2 Materials and methods

### 2.1 Plant materials and field experiments

Four DH populations (TOC1, TOC2, TOC3 and TOC4) were constructed as previously method described (Chaikam et al., 2019; Du et al., 2020). The eight inbred parental lines exhibiting the variation in TOC (**Table 1**) were belonged to Maize Yufeng Biotechnology LLC (Beijing, China) and selected as elite inbred lines used for optimizing grain nutritional quality breeding program. Parents of TOC1 and TOC2 belong to maize Lancaste germplasm, and parents of TOC3 and TOC4 belong to Reid Yellow Dent germplasm. The populations (TOC1, TOC2, TOC3 and TOC4) including 123, 129, 281 and 160 lines, respectively (**Table 1**). Each population with its parents were planted in 2021 at Liaoning province, China (LN, 40^°^`82′N, 123^°^56′E) with three replication blocks. All lines were planted in a single row plot with the length of 150 cm and 60 cm using a complete randomized block design under natural field conditions. All plants were self-pollinated and kernels from middle part of three well-grown ears were harvested and dried for oil measurement. We declare that all the collections of plant and seed specimens related to this study were performed in accordance with the relevant guidelines and regulations by Ministry of Agriculture (MOA) of the People’s Republic of China.

**Table 1:** Phenotypic performance, variance, and broad-sense heritability of TOC in the four DH populations.

Trait ^a^	Populations							
	TOC1		TOC2		TOC3		TOC4	
Parents								
**means ± SD (%)**	KB717001	4.14 ± 0.17	KB717001	4.14 ± 0.17	AJ519002	4.30 ± 0.10	AJ519004	4.43 ± 0.02
	KB519009	3.50 ± 0.15	KB719010	3.16 ± 0.05	AJ519001	4.90 ± 0.09	AJ519006	4.95 ± 0.09
***p* value** ^**b**^	0.008**		0.006**		0.002**		0.007**	
DHs								
**Size**	123		129		281		160	
**means ± SD (%)**	4.57 ± 0.41		4.42 ± 0.40		4.50 ± 0.42		5.02 ± 0.41	
**Range (%)**	3.64 - 5.58		3.59 - 5.48		3.10 - 5.42		4.06 - 6.13	
**σ*_g_* ^2 c^**	0.205		0.183		0.186		0.168	
**σ*_e_* ^2 d^**	0.027		0.059		0.023		0.009	
**σ*_ϵ_* ^2 e^**	0.126		0.085		0.274		0.197	
***h^2^* (%)** ^**f**^	83.00%		86.60%		67.00%		71.80%	

a TOC;

b P value based on a t-test evaluating two parental lines;

c genetic variance;

d environmental variance;

e residual variance^;^

f broad-sense heritability (h^2^);

** p ≤ 0.01.

### 2.2 Evaluation of oil content and statistical analysis of phenotypic data

Near infrared reflectance (NIR) spectrometer (DA 7250, Perten Instruments Inc., Sweden) was used to measure TOC in maize kernels as previously described with a few modifications (Chen and Hu, 2017). The reflectance spectra were collected in a range of 400 to 2500 nm with 10-nm intervals in the NIR region. A minimum of 50 kernels per sample was scanned three times and the average was taken as final phenotypic value.

All statistical analyses were performed by using R Version 4.0.1 (www.R-project.org) as previously described (Zhang et al., 2021; Zhang et al., 2022). The R ‘AOV’ function was used to estimate the variances of TOC. The model for the variance analysis was as following: y = μ + α_g_ + β_e_ + ϵ, where α_g_ is the effect of the g^th^ line, β_e_ is the effect of the e^th^ environment, and ϵ is the error. The effects in the model were defined by random. The broad-sense heritability (*h^2^*) analyzed in the populations was calculated according to Knapp et al., 1985. The formula was *h^2^ =* σ*_g_* ^2^ */*(σ*_g_* ^2^ *+* σ*_ϵ_* ^2^/*e*), where ${\text{σ}}_{g}^{2}$ is the genetic variance, ${\text{σ}}_{\varepsilon }^{2}$ is the residual error, and *e* is the number of environments. The best linear unbiased predictor (BLUP) value of each line was calculated as: y_ij_ = μ + e_i_ + f_j_ + ϵ_ij_, where y_ij_ is the phenotypic value of individual j in environment i, μ is the grand mean, e_i_ is the effect of different environments, f_j_ is the genetic effect, and ϵ_ij_ is the random error. The grand mean was fitted as a fixed effect, and genotype and environment were considered random effects (Wang et al., 2015). All of these variances were estimated using the ‘LME4’ R package. The BLUP values were used for phenotypic description statistics and QTL analysis.

### 2.3 Genotyping and constructing genetic linkage map

The four DH populations with their parents were genotyped using the GenoBaits Maize 1K marker panel (Mol Breeding Biotechnology Co., Ltd., Shijiazhuang, China). A total of 4,589 SNP markers were identified on the basis of genotyping by target sequencing platform (Guo et al., 2019). The minor allele frequency (MAF) and missing rate were estimated in each population and the SNPs with MAF < 0.1 or missing rate > 0.6 were filtered out. After quality control, the polymorphic SNPs between two parental lines were used to construct the genetic linkage maps using the R/qtl package functions est.rf and est.map (Broman et al., 2003) with the kosambi mapping method.

### 2.4 QTL mapping

Composite interval mapping (CIM) method followed by multiple QTL mapping analysis was performed using Windows QTL Cartographer 2.5 and R language (Wang et al., 2010a). The whole genome was scanned at every 1.0 cM interval with a window size of 10 cM. A forward and backward stepwise regression with five controlling markers was conducted to control background from flanking markers. The empirical logarithm of the odds (LOD) threshold was calculated using 1,000 permutations at a significance level of *p* = 0.05 (Churchill and Doerge, 1994). These threshold LOD values were in a range of 2.76 to 3.06 in four DH populations. QTLs with LOD value greater than the threshold were considered for further analysis. With the 1.5-LOD support interval method, the confidence interval for each QTL position was estimated (Lander and Botstein, 1989). The additive × additive epistatic interactions was performed by “IM-EPI” method in IciMapping Version 4.2.

### 2.5 Gene annotation

QTLs were delimited to a single peak bin interval based on bin map. The protein-coding genes within intervals were listed according to MaizeGDB database (V2). Each of the corresponding gene were annotated by performing BLASTP searches at the NCBI (blast.ncbi.nlm.nih.gov/Blast.cgi).

## 3 Results

### 3.1 Phenotypic variation and heritability of TOC in maize kernel

**Figure 1:**
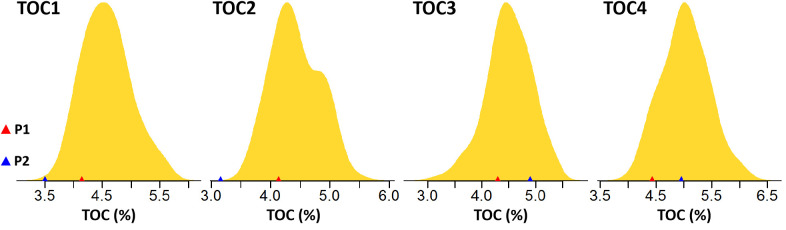
Phenotypic variation in TOC in the four DH populations. The *x*-axis showed the TOC and the triangle color indicated the TOC in parents.

Four DH populations, TOC1-TOC4 were developed from eight inbred lines (TOC with a range of 3.16-4.95%). Each population contained 123-281 lines, respectively (**Table 1**). Within each DH population, TOC exhibited a continuously and approximately normal distribution, which is the typical characteristic of quantitative trait (**Figure 1** and **Table 1**). Analysis of variance (ANOVA) revealed that the genotype variance was greater than environmental variance in all populations (**Table 1**), indicating that phenotypic variations were mainly controlled by genetic factors. Broad-sense heritability estimates were calculated and showed high for TOC1 and TOC2 populations (83.00-86.60%), and moderate for TOC3 and TOC4 populations (67.00-71.80%) (**Table 1**). The moderate to high heritability indicated that most of TOC variations in these DH populations were genetically controlled and suitable for further QTL mapping.

### 3.2 Genotyping and genetic linkage map

A GenoBaits Maize 1K SNP marker panel was used for genotyping all DH lines in the four populations. After quality control, a total of 1,217, 575, 1,022 and 1,039 polymorphic SNPs were identified for TOC1-TOC4 populations, respectively. These high-fidelity SNPs were used to construct the genetic linkage map with the missing rate in most lines less than 2% (**Figure S1**). In total, 925.92, 684.23, 860.81 and 836.67 cM genetic distances spanned in four linkage maps (**Figure S2**), and the average genetic distance between every two adjacent markers was 0.77, 1.21, 0.85, and 0.81 cM in each DH population, respectively (**Table S1**).

### 3.3 Identification of QTLs for TOC in four DH populations

**Figure 2:**
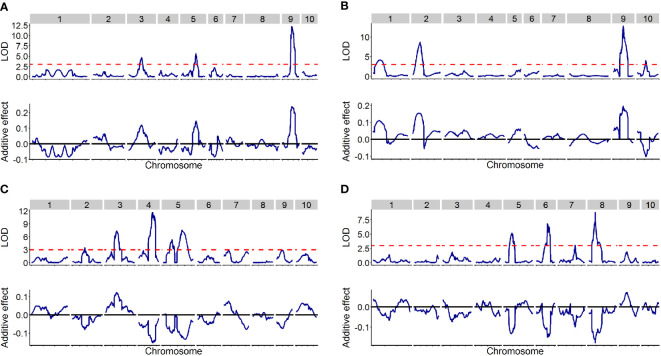
The distribution of QTLs across the entire genome in the four DH populations. The upper of each picture displayed LOD score (*y*-axis) against the physical position (*x*-axis) of markers, while the bottom of the picture displayed additive effect (*y*-axis) against the physical position (*x*-axis) of markers. **(A–D)** designated TOC1, TOC2, TOC3 and TOC4, respectively.

A total of 16 QTLs were identified with a LOD threshold of above 3.00 at the 0.05 significance level (**Table 2** and **Figure 2**). Among them, 3, 4, 5 and 4 QTLs were detected in TOC1, TOC2, TOC3 and TOC4, respectively. The average genetic intervals of these QTLs was 82.69 cM in a range of 36.56-125.29 cM. The average physical interval was 102.58 Mb in a range of 11.96-232.42 Mb. The contribution to phenotypic variation for each population ranged from 40.99 (TOC3) to 62.05% (TOC2) with an average of 51.10%. The explained phenotypic variation were less than broad-sense heritability (**Tables 1**, **2**), suggesting that only part of QTLs have been detected in these bi-parent populations.

**Table 2:** Individual QTL for TOC in the four DH populations.

Populations	QTL	Chr.^a^	G-Peak (cM)^b^	P-Peak (Mb)_V4^c^	G-Range (cM)^d^	P-Range (Mb)_V4^e^	LOD	PVE%^f^	Add.^g^	Parent ^h^+	PVE(%) -ALL^i^
TOC1	*qOC-1-1*	3	48.55	162.54	41.05-52.23	112.37-169.00	4.68	7.50	0.12	KB717001	55.82
	*qOC-1-2*	5	53.92	193.53	50.84-58.39	191.47-199.14	5.61	11.64	0.15	KB717001	
	*qOC-1-3*	9	34.02	125.24	31.10-41.66	113.85-143.02	12.24	30.84	0.24	KB717001	
TOC2	*qOC-2-1*	1	19.15	12.72	8.15-29.13	12.72-26.18	4.17	7.26	0.11	KB717001	62.05
	*qOC-2-2*	2	20.42	30.39	13.01-24.42	11.17-30.39	8.67	13.53	0.15	KB717001	
	*qOC-2-3*	9	27.23	129.70	25.45-28.83	122.00-130.80	12.80	21.74	0.20	KB717001	
	*qOC-2-4*	10	23.05	55.99	23.05-24.63	55.99-79.47	4.03	5.72	-0.10	KB719010	
TOC3	*qOC-3-1*	2	42.18	58.26	40.75-42.18	46.13-58.26	3.49	3.49	-0.08	AJ519001	40.99
	*qOC-3-2*	3	37.82	22.64	29.65-44.47	11.58-149.70	7.37	8.39	0.12	AJ519002	
	*qOC-3-3*	4	47.22	232.42	43.03-56.33	196.02-241.81	11.71	12.99	-0.15	AJ519001	
	*qOC-3-4*	5	30.81	43.18	23.31-38.99	15.74-85.58	5.33	5.41	-0.10	AJ519001	
	*qOC-3-5*	5	65.51	202.27	58.23-79.97	188.27-207.38	7.58	8.26	-0.12	AJ519001	
TOC4	*qOC-4-1*	5	24.43	11.96	15.65-31.84	6.09-20.79	5.12	8.84	-0.13	AJ519006	45.54
	*qOC-4-2*	6	36.45	131.71	35.42-45.25	129.11-140.52	6.83	13.05	-0.15	AJ519006	
	*qOC-4-3*	7	54.69	165.51	54.69-54.69	146.02-168.32	3.04	5.07	-0.10	AJ519006	
	*qOC-4-4*	8	21.13	63.28	19.12-22.76	10.65-65.48	8.82	16.20	-0.18	AJ519006	

a Chromosome;

b Genetic position in centimorgans (cM) of QTL with the highest LOD;

c Physical position of QTL based on the B73 reference sequence (V4);

d Genetic position range in centimorgans (cM) of QTL with the highest LOD;

e Physical position range of QTL based on the B73 reference sequence (V4);

f Percentage of the phenotypic variation explained by the additive effect of QTL;

g Additive effect of QTL;

h which parental allele increased the TOC;

i Percentage of the phenotypic variation explained by the additive effect of all QTL.

In TOC1, three QTLs (*qOC-1-1, qOC-1-2 and qOC-1-3*) distributed on chromosome 3, 5 and 9. The QTL, *qOC-1-3*, with the largest effect (30.84% of the phenotypic variation) was located on chromosome 9. The parental KB717001 allele at this locus had an additive effect of 0.24% for increased oil content. The second QTL *qOC-1-2* was located on chromosome 5, and explained 11.64% of phenotypic variance with an additive effect of 0.15%. *qOC-1-1* on chromosome 3 explained 7.50% of the phenotypic variance and considered as a minor QTL. The parent KB717001 allele at all of mapped loci had increasing effects for TOC.

In TOC2, four QTLs (*qOC-2-1*, *qOC-2-2*, *qOC-2-3* and *qOC-2-4*) were identified and accounted for 62.50% of the total phenotypic variance. One major QTL *qOC-2-3* located on chromosome 9 and contributed to 21.74% of the explained phenotypic variance. The second QTL *qOC-2-2* on chromosome 2 explained 13.53% of phenotypic variance with an additive effect of 0.15%. The *qOC-2-1* and *qOC-2-4* explained 5.72% and 7.26% of the phenotypic variance, respectively The parent KB717001 allele increased the TOC for *qOC-2-1*, *qOC-2-2* and *qOC-2-3*, but decreased the TOC for *qOC-2-4.*

In TOC3, a total of five QTLs (*qOC-3-1*, *qOC-3-2*, *qOC-3-3*, *qOC-3-4* and *qOC-3-5*) were detected and explained 40.99% of the total phenotypic variance. *qOC-3-3* on chromosome 4 was the major QTL explaining phenotypic variation of 12.99% with an additive effect of 0.15%. The parent AJ519002 allele at *qOC-3-2* increased the TOC, whereas the parent AJ519001 allele at other QTLs increased the TOC.

In TOC4, a total of four QTLs were identified (*qOC-4-1*, *qOC-4-2*, *qOC-4-3* and *qOC-4-4*) and accounted for 45.54% of the total phenotypic variance. *qOC-4-2* on chromosome 6 and *qOC-4-3* on chromosome 8 were two major QTLs explaining the phenotypic variation of 13.05% and 16.20%, respectively. *qOC-4-1* and *qOC-4-3* were two minor QTLs explaining 8.84% and 5.07% phenotypic variation, respectively. The parent AJ519006 allele at all these QTLs increased the TOC.

### 3.4 Genetic overlap of QTLs in the four DH populations with other populations

**Figure 3:**
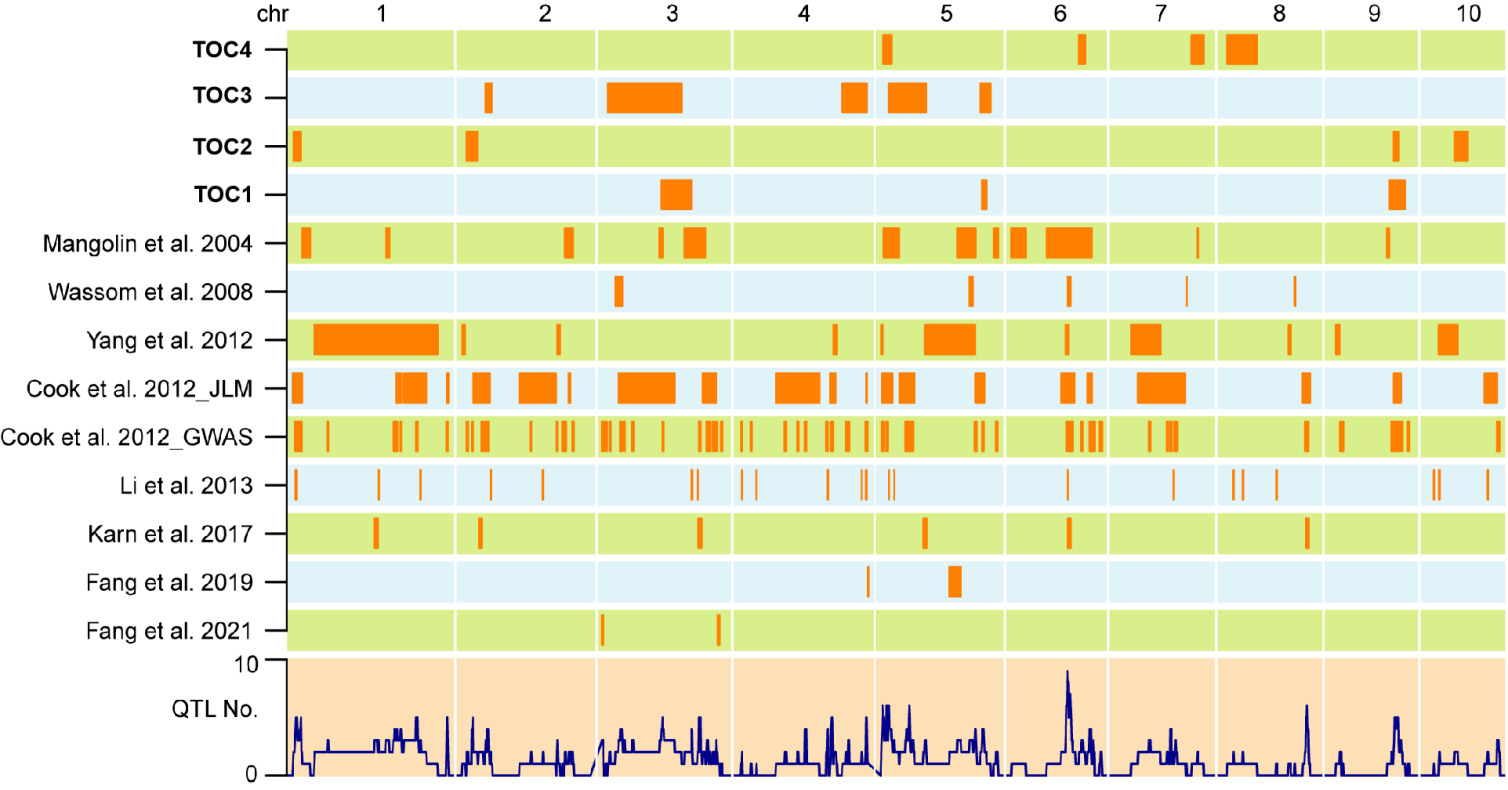
Co-localization of TOC QTLs in maize kernels identified in the present and previous studies. The QTLs identified in this study were represented on top. QTLs detected in previous studies were displayed in the form of references. The lower layer showed the number of detected QTLs.

Several overlapped QTLs regions were detected across the four populations, including a 37.32 Mb overlap between *qOC-1-1* and *qOC-3-2*, and a 5.05 Mb overlap between *qOC-3-4* and *qOC-4-1* (**Figure 3**). Moreover, *qOC-1-2* and *qOC-2-3* located within *qOC-3-5* and *qOC-1-3*, respectively (**Figure 3**).

To investigate whether these newly-identified QTLs shared across different genetic background, we compared their genomic locations with QTLs related to oil traits from the other eight previous studies (Mangolin et al., 2004; Wassom et al., 2008a; Wassom et al., 2008b; Wang et al., 2010b; Cook et al., 2012; Yang et al., 2012; Li et al., 2013; Yang et al., 2016; Karn et al., 2017; Fang et al., 2020 and Fang et al., 2021). A total of 56 genomic regions related to oil synthesis and accumulations were identified to be overlapped with QTLs in our four DH populations (**Figure 3**). These results indicated that although unique and specific QTLs were detected in each population, some genetic loci may have common effects on TOC among different types of populations.

## 4 Discussion

### 4.1 QTL mapping precision

The genetic architecture of a quantitative trait consists of a set of parameters that explain the genetic component of trait variation within or among populations (Laurie et al., 2004). These parameters include the number of QTL affecting the trait, their locations in the genome, the frequencies of alternative genotypes segregating at the QTL, the pattern of linkage disequilibria among QTL, and the magnitudes of additive, dominance, and epistatic effects (Laurie et al., 2004). Different types of populations used in QTL mapping tend to vary with two main characteristics: (1) their ability to capture genetic diversity, and (2) their power to detect QTL of small effect (Odell et al., 2022). The advantages of DH populations are the capability of removing any residual heterozygosity to ensure genetically identical replicates and increasing selection response by stabilizing heritability of various traits during perse and test cross evaluation (Bordes et al., 2006; Gallais and Bordes, 2007; Mayor and Bernardo, 2009; Odell et al., 2022).

SNP markers are the most frequent variations in genomes and the application of SNP markers in plant breeding has guaranteed the precision of QTL mapping and genetic analysis (Bhattramakki et al., 2002; Mammadov et al., 2012; Flutre et al., 2022; Kaur et al., 2022). By conditioning linked markers in the test, the sensitivity of the test statistic to the position of individual QTLs is increased, and the precision of QTL mapping can be improved (Zeng, 1994). Subsequently, with the development of sequencing technology, an increasing number of molecular markers have been applied to QTL mapping, which greatly improves the accuracy of QTL mapping (Schnable et al., 2009; Chia et al., 2012; Bukowski et al., 2018; Fang et al., 2021). In this study, a total of 16 QTLs were found and distributed across all ten chromosomes. 13 QTLs spanned physical intervals of less than 50 Mb, and two span less than 10 Mb. Thus, the resolution in this study is considerably improved because of the large number of markers and the appropriate population type. The resolution is probably on the order of 2-3 cM, since pairs of markers any farther apart rarely have substantial levels of linkage disequilibrium (Laurie et al., 2004).

### 4.2 Genetic basis of TOC in our DH populations

Within the four DH populations, a broad range of phenotypic variation with normal distribution was observed for TOC with transgressive segregation, indicating quantitative genetic control (**Figure 1**). The identification of loci controlling oil-related traits should contribute to a better understanding of oil synthesis and storage in maize kernels. The genetic analysis indicated TOC is highly heritable and the heritability (67.00-86.60%) is fairly high in all populations, indicating of superior genetic effect on TOC in DH populations. The high heritability estimates are very favorable for detecting marker-trait associations (Laurie et al., 2004). Among the 16 detected QTLs controlling TOC, 11 QTLs were identified as the major QTLs with the explaining phenotypic variation larger than 10%. Especially *qOC-1-3* with the largest effect (30.84% of the phenotypic variance) and *qOC-2-3* with the second largest effect (21.74% of the phenotypic variance) were located on chromosome 9. These region have been chosen as our primary QTL for further study because of the higher contribution. The parent allele at this locus had an additive effect of 0.20-0.24% for increased TOC. An additional seven QTLs were identified on chromosomes 2, 4, 5, 6 and 8, explaining between 11.64 and 16.20% of the phenotypic variation. The other minor QTLs each explained 3.49-8.39% of the phenotypic variance with moderate additive effects on TOC. In addition, except for environment variation, none of QTLs were shared by all DH populations, reflecting the complexity of TOC regulation in diverse maize populations. These results indicated that oil content is controlled by a few large-effect QTLs, together with a large number of minor-effect QTLs (Dudley, 1977; Laurie et al., 2004).

Results of QTL detection derived from different studies may exhibit consistency to a certain degree across different germplasms or genetic backgrounds and environments. For instance, the largest and second effective QTL *qOC-1-3* and *qOC-2-3* was located in the QTL *m240* with a 29.17 Mb and 8.81 Mb overlap interval length, respectively, which was related to maize TOC in RIL population (Cook et al., 2012). *qOC-3-4* co-localized with *koc5b* associated to the kernel oil content in a F_2:3_ tropical maize population (Mangolin et al., 2004). According to Li et al. (2013), the QTL *qOC-2-1*, *qOC-3-1*, *qOC-3-3*, *qOC-4-1* and *qOC-4-1* more or less co-localized with the QTLs controlling protein and TOC simultaneously and might affect protein and TOC in opposite directions (Li et al., 2013). These results suggested that increases in grain TOC might be associated with increases in grain protein content, both traits could be improved simultaneously. Congruence in QTLs detected in this study with previous reports indicates the robustness of our results. Moreover, these QTLs definitely worth conducting further research on this QTL *via* NILs, fine mapping, molecular marker-assisted selection (MAS) and ultimate cloning.

### 4.3 Importance of QTLs relevant to TOC in maize genetic and breeding

**Figure 4:**
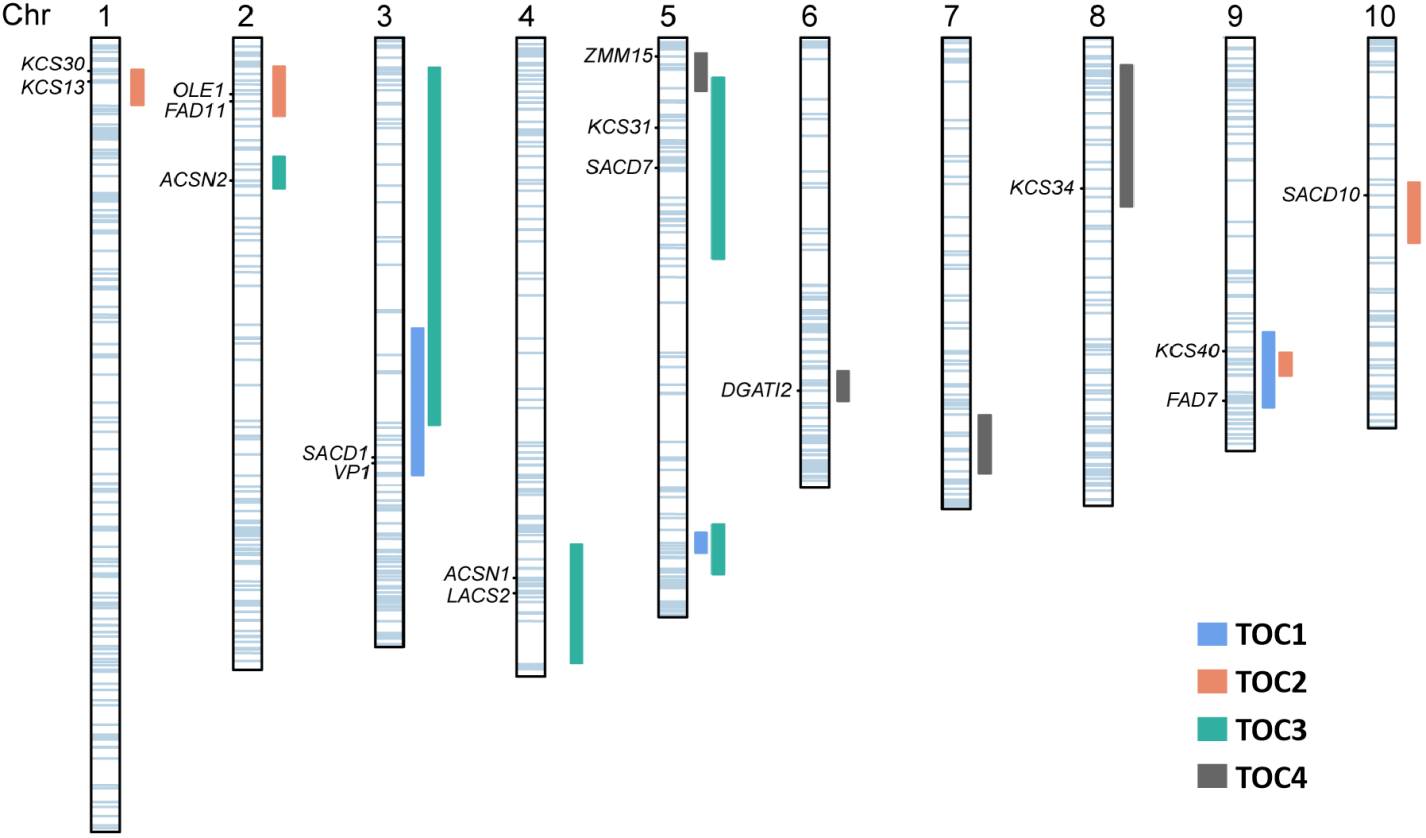
Association of candidate genes with kernel oil QTLs. The QTLs identified in four DH populations are represented as vertical rectangles of different colors next to each chromosome. The horizontal light blue bars on each chromosome show the positions of the 189 identified genes. The left labels denote known genes that co-localized with the QTLs.

Oil in maize kernels mainly exists in the form of triacylglycerol (TAG), which composed of fatty acids and glycerol (Du et al., 2016; Zhang et al., 2019). Maize oil mainly accumulates in the embryo, and the fatty acids are typically comprised of approximately 11% palmitic acid (C16:0), 2% stearic acid (C18:0), 24% oleic acid (C18:1), 62% linoleic acid (C18:2), and 1% linolenic acid (C18:3) (Lambert, 2001). The quality and utilization of maize oil is determined by their fatty acid composition (Du et al., 2016). Saturated fatty acids, such as palmitic (C16:0) and stearic acids (C18:0), are stable and tolerant to heat and oxidation (Hu et al., 1997). Certain unsaturated fatty acids, such as oleic (C18:1), linoleic (C18:2), and linolenic (C18:3) acids, are beneficial to human health but susceptible to heat and oxidation (Hu et al., 1997). Biosynthesis of storage oil in plant seeds is complex and involved in multitudinous physiological and biochemical processes (Ohlrogge and Browse, 1995; Liu et al., 2008; Zhang et al., 2009; Guo et al., 2013; Dong et al., 2015; Glowinski and Flint-Garcia, 2018; Zhang et al., 2018). The co-location analysis of candidate genes underlying QTLs associated with related trait could provide information about functional relationships between gene expression and some QTLs of the complex biosynthesis pathway (Prioul et al., 1997; Thévenot et al., 2005). In our study, of 189 genes involved in the fatty acid biochemical processes, including 17 well-known genes encoding key enzymes in maize lipid synthesis and metabolism, were located within QTL intervals (**Figure 4** and **Table S2**).

The genes related to the TAG synthesis pathway are key regulatory factors in the accumulation process of TOC in corn (Zhang et al., 2019). Comparison of the positions of candidate genes and QTL was a suitable strategy to investigate the molecular basis of quantitative traits. Additionally, the positioned candidate genes can be used to develop functional markers for increasing selection efficiency by marker-assisted selection in plant breeding (Andersen and Lübberstedt, 2003). Five KCS genes encoding *β*-ketoacyl CoA synthase isozymes in *qOC-1-3*, *qOC-2-1*, *qOC-3-4* and *qOC-4-4* are mainly involved in the process of elongation of the C16:0- and C18:0-CoAs into very-long-chain fatty acids (VLCFAs) (Gonzales-Vigil et al., 2017). The maize isozymes reflected differences in the enzymatic capability to elongate fatty acids (Stenback et al., 2022). The *FAD* genes in *qOC-1-3* and *qOC-2-2* were identified as fatty acid desaturase-coding and are responsible for the production of trienoic fatty acids by unsaturation at the ω-3 position and the cDNAs corresponding to the loci have been isolated (Ohlrogge and Browse, 1995; Gao et al., 2015; Zhao et al., 2019). Stearoyl-acyl carrier protein desaturases (SACD) encoded by the genes in *qOC-1-1*, *qOC-2-4* and *qOC-3-4* are the key enzymes that converts stearic acid to oleic acid by introducing the first double bond into stearoyl-ACP between carbons 9 and 10 (Asamizu et al., 1998; Liu et al., 2009). These enzymes are significantly more abundant in expression in high-oil maize than in normal maize, not only at the mRNA and protein levels, but also at the product level (Liu et al., 2009). *LACS2* in *qOC-3-3* encoded the long-chain acyl-CoA synthetase (LACS), which plays key roles in activating fatty acids to fatty acyl-CoA thioesters and then further involved in lipid synthesis and fatty acid catabolism (Lü et al., 2009; Zhao et al., 2010; Jessen et al., 2011 and Jessen et al., 2015). TAG biosynthesis involves three consequential acylation steps of a glycerol backbone *via* the Kennedy pathway (Ohlrogge and Browse, 1995; Iskandarov et al., 2017; Müller and Ischebeck, 2018). The process starts with the acylation of glycerol-3-phosphate (G3P) by glycerol-3-phosphate acyltransferase (GPAT) and lysophosphatidic acid acyltransferase (LPAAT), and finalized by diacylglycerol acyltransferase (DGAT), which catalyzes the last acylation step of the pathway (Ohlrogge and Browse, 1995). The high-oil QTL (*qHO6*) affecting maize seed oil and oleic-acid contents encodes DGAT1-2 (Zheng et al., 2008; Yang et al., 2010; Hao et al., 2014). The gene *GPAT12* in our study was also detected on chromosome 6 and showed 96% identities with *DGAT1-2* (Zm00001d036982), which indicated that GPAT12 may be one of DGAT isozymes. The seed oils are packaged in spherical intracellular oil bodies, which have a TAG matrix surrounded by a layer of phospholipids embedded with unique and abundant proteins termed oleosins (Lee and Huang, 1994). Oleosins interact with the surface phospholipids and matrix triacylglycerols to form a stable amphipathic layer on the surface of the oil body and possibly act as recognition signals for the binding of lipase during germination (Lee and Huang, 1994; Lee et al., 1995; Ting et al., 1996). It suggested that *OLE1* in *qOC-2-2* was an important gene that would facilitate lipase action during germination. The above analysis suggested that the QTLs in this study were related to a series of genes encoding key enzymes relevant to oil content and lipid metabolism. Especially, *qOC-4-2* contained a DGAT1-2 homologous protein coding gene and had no common region with *qHO6* which was the major oil content QTL (Cook et al., 2012). Therefore, these QTLs will pave a path to explore molecular markers and offer prospective routes to improve maize oil content through molecular marker-assisted selection in maize breeding program.

## 5 Conclusion

In this study, four DH populations were constructed for genetic analysis of kernel TOC and the TOC exhibited continuously and approximately normal distribution in all populations. Six major and ten minor effect QTLs were identified based on the genetic linkage map with LOD threshold of 3.00 and accounted for 3.49-30.84% of oil variation. The result was consistent with Yang et al., 2010 that OC in maize kernel is a complex quantitative trait and controlled by a few large-effect QTLs and numerous minor QTLs. Besides, 17 well-known genes involved in fatty acid synthesis and metabolic pathway were located within QTL intervals. This information provides insight that will help to further understanding of genetic variation in TOC in maize kernels and will thus enhance the feasibility of cloning QTL, lay the foundation to explore candidate genes associated with maize kernel TOC.

## Data availability statement

The original contributions presented in the study are publicly available. This data can be found here: https://doi.org/10.6084/m9.figshare.22152380.v1.

## Author contributions

HL and CZ conceived and designed the experiments. XZ, HG, HW, CD, JW, BP, and JL performed the research. MW analysed the data. XZ and MW wrote the manuscript. All authors contributed to the article and approved the submitted version.
