# Supplementary material for: Genetic dissection of QTLs for oil content in four maize DH populations
Source: Front Plant Sci. 2023 Apr 12;14:1174985. doi: 10.3389/fpls.2023.1174985 (PMC10130369; doi:10.3389/fpls.2023.1174985)
Supplement: Supplementary file 1 [file DataSheet_1.docx]

Supplementary Material

## Supplementary Figures

**A**

**B**


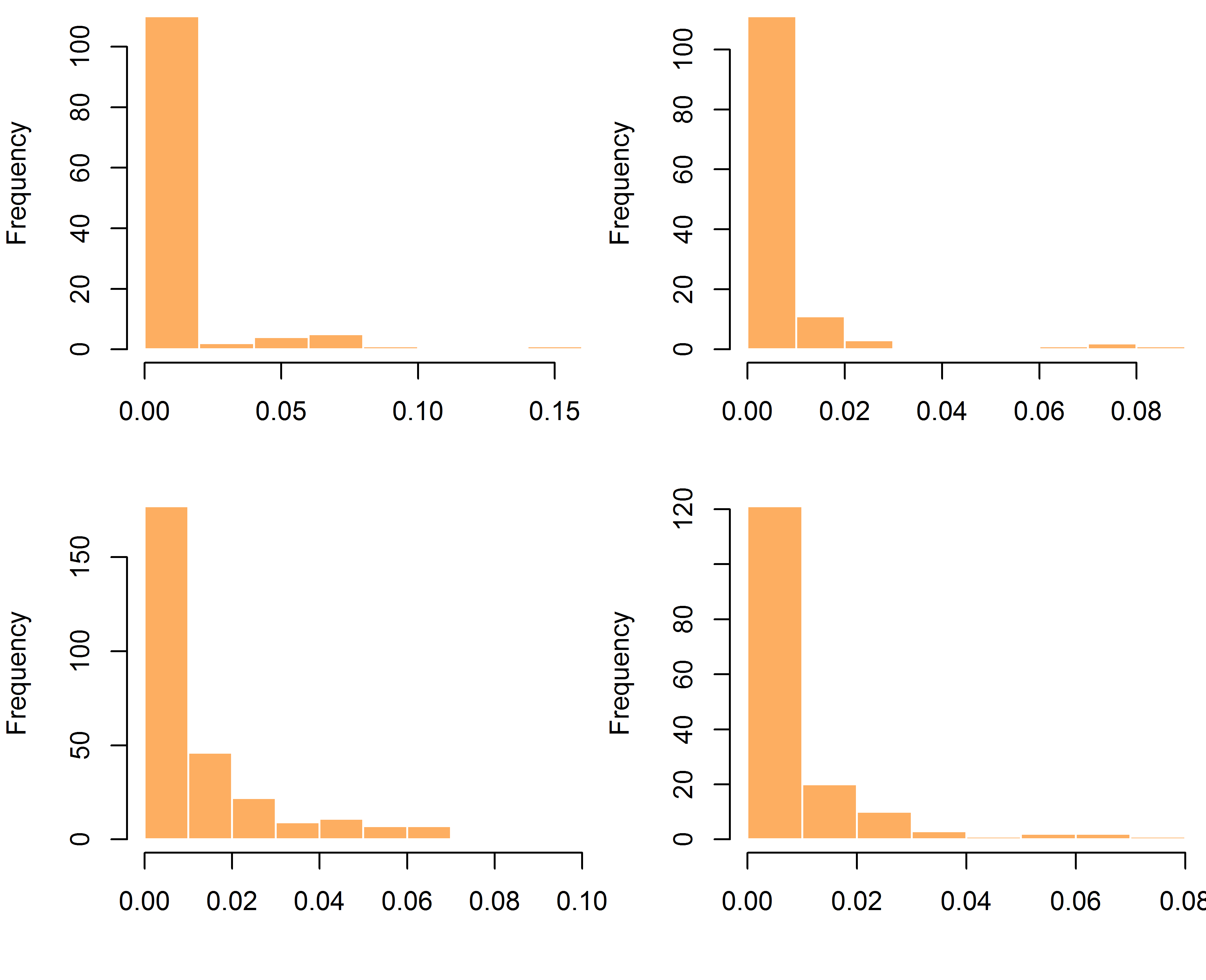


**D**

**C**

**Supplementary Figure 1.** Distribution of the missing rate for SNP in each line. (A), (B), (C) and (D) designated TOC1, TOC2, TOC3 and TOC4, respectively.


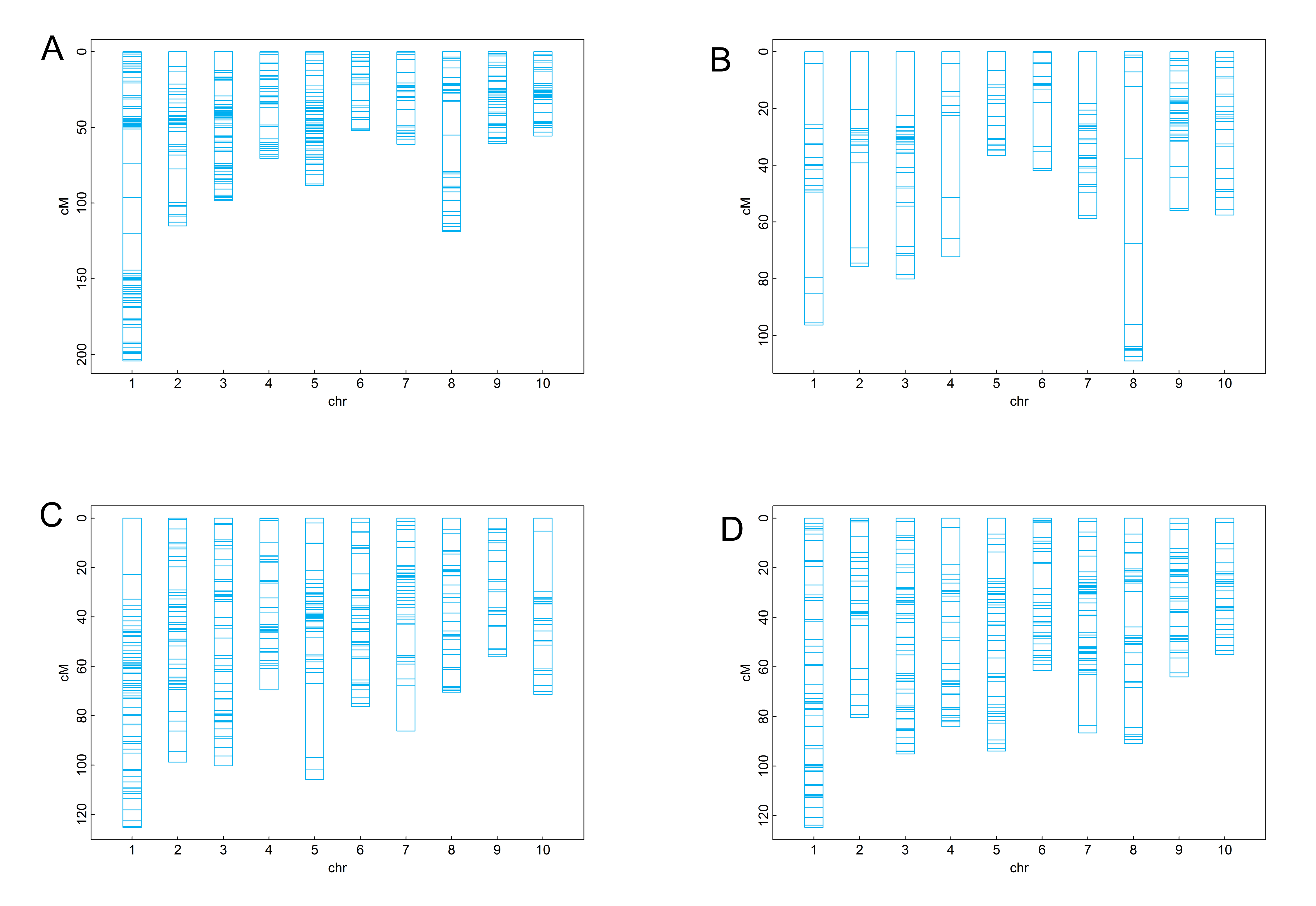


**Supplementary Figure 2.** Linkage maps of four DH populations. The horizontal light blue bars on each chromosome showed the genetic position of each SNP. (A), (B), (C) and (D) designated TOC1, TOC2, TOC3 and TOC4, respectively.
